# Video Assisted Thoracic Surgery for Indeterminate Pulmonary Nodules

**DOI:** 10.1155/DTE.1.25

**Published:** 1994

**Authors:** Janet L. Albright, Rick I. MacArthur, Julie A. Swain, Alan T. Tran, Alex G. Little

**Affiliations:** 1 University of Nevada School of Medicine Department of Surgery Kansas city Kansas USA; 2 Department of Surgery University of Nevada School of Medicine 2040 West Charleston Boulevard Suite 601 Las Vegas Nevada 89102 USA

## Abstract

The approach to patients with indeterminate pulmonary nodules is poorly defined. Should every pulmonary nodule be biopsied, is needle biopsy adequate, and other questions are challenges. Video assisted thoracic surgery or thoracoscopy has added a new diagnostic possibility which is evaluated in this paper. Fifty-five patients underwent thoracoscopy for diagnosis of a solitary pulmonary nodule. There were few complications and mortality was zero. A definitive diagnosis was obtained in all patients, although one required a second thoracoscopic wedge resection and 10 required conversion to an open thoracotomy.

As discussed in the paper, thoracoscopy provides the opportunity for safely establishing a definitive diagnosis in all patients with solitary nodules and the authors believe will become a standard part of the evaluation of these patients.


*Diagnostic and Therapeutic Endoscopy*, Vol. 1, pp. 25–28
Reprints available directly from the publisher
Photocopying permitted by license only

© 1994 Harwood Academic Publishers GmbH
Printed in Malaysia

### Video Assisted Thoracic Surgery for Indeterminate Pulmonary Nodules

JANET L. ALBRIGHT, RICK I. MACARTHUR, JULIE A. SWAIN, ALAN T. TRAN and ALEX G. LITTLE

*University of Nevada School of Medicine, Department of Surgery, Kansas City, Kansas*

*(Received October 18, 1993; in final form January 31, 1994)*

The approach to patients with indeterminate pulmonary nodules is poorly defined. Should every pulmonary nodule be biopsied, is needle biopsy adequate, and other questions are challenges. Video assisted thoracic surgery or thoracoscopy has added a new diagnostic possibility which is evaluated in this paper. Fifty-five patients underwent thoracoscopy for diagnosis of a solitary pulmonary nodule. There were few complications and mortality was zero. A definitive diagnosis was obtained in all patients, although one required a second thoracoscopic wedge resection and 10 required conversion to an open thoracotomy.

As discussed in the paper, thoracoscopy provides the opportunity for safely establishing a definitive diagnosis in all patients with solitary nodules and the authors believe will become a standard part of the evaluation of these patients.

**KEY WORDS:** lung cancer, thoracoscopy, solitary pulmonary nodule

### INTRODUCTION

The recent surge of popularity of endoscopic or minimally invasive surgery has brought carcinoma of the lung, the most common visceral cancer in the United States, into the arena of thoracoscopy or video assisted thoracic surgery (VATS), but its role is not yet defined. A possible therapeutic role with performance of wedge resection as a definitive treatment of small lung tumors is not promising, as local recurrence and survival rates indicate that any resection less than lobar is inadequate for cure of lung cancer (Ginsburg, personal communication). Until anatomic resection such as lobectomy and pneumonectomy are established as standard thoracoscopic procedures, it appears that possible routine roles for thoracoscopy in the care of lung cancer patients are diagnosis and/or intrathoracic staging. We, therefore, reviewed our experience with video assisted resection of pulmonary abnormalities to assess the diagnostic role of VATS. A particular interest was the possibility that VATS might improve early diagnosis of nonsmall cell lung cancer. Despite excitement over multimodality therapeutic approaches, little progress has been made in the improvement of overall survival of lung cancer patients. Earlier detection would presumably identify patients with an earlier stage of disease, thus improving the possibility of efficacious treatment and cure.

### MATERIALS AND METHODS

VATS is performed with a standard 0-degree viewing camera placed through a 10-mm port. Additional ports of varying size are placed for access as needed. Multiple retracting and/or grasping instruments are employed and a 30-mm endostapler is used to perform the wedge resections. We utilized one-lung anesthesia with a double lumen endotracheal tube. $CO_2$ insufflation for lung collapse to aid in visualization has not been required.

The patient is positioned in a lateral decubitus position and initial access is gained through a midaxillary port, inserted after finger inspection to assure the absence of local adhesions between lung and pleura. After deflation of the ipsilateral lung, the thoracoscope is introduced to visualize the insertion of all subsequent ports. Adhesions are divided with electrocautery. Ports are placed according to the location of the parenchyma to be biopsied with the goals of having them spread apart rather than close to-

Address for correspondence: Alex G. Little, M.D., Department of Surgery, University of Nevada School of Medicine, 2040 West Charleston Boulevard, Suite 601, Las Vegas, Nevada 89102.

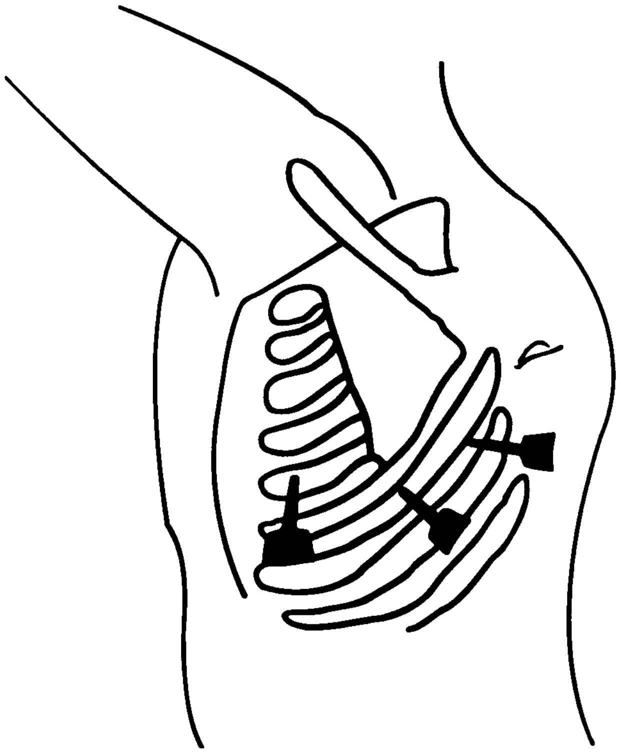

**Figure 1** This schematic drawing illustrates a typical distribution of ports for access during VATS.

gether and having them oriented with rather than toward the camera which can lead to an awkward "fencing" scenario. A typical distribution is shown in Figure 1. It is occasionally necessary to enlarge a port access incision to allow finger palpation of the tissue and/or to facilitate tissue removal.

**Table 1**

| *Clinical Indication* | *Number* | *Final Diagnosis* | *Number* |
|---|---|---|---|
| Solitary Nodule | 45 | Small cell carcinoma | 1 |
| | | Large cell carcinoma | 2 |
| | | Squamous cell carcinoma | 3 |
| | | Bronchoalveolar carcinoma | 1 |
| | | Adenocarcinoma | 9 |
| | | Lymphoma | 1 |
| | | Granuloma | 11 |
| | | Histoplasmosis | 10 |
| | | Coccidiomycosis | 1 |
| | | Pulmonary infarction | 3 |
| | | Inflammation/anthracosis | 2 |
| | | Atelectasis | 1 |
| Recurrence/Metastatic Work-up | 10 | Confirmation | 6 |
| | | Squamous cell carcinoma | 1 |
| | | Large cell carcinoma | 1 |
| | | Alveolitis | 1 |
| | | Carcinoid | 1 |

From April, 1991 through November, 1992, thoracoscopy was performed on 55 patients for diagnosis of pulmonary nodules. Our indications for VATS are listed in Table 1. Another 9 patients, who are not included in this paper, underwent thoracoscopic lung biopsy for diagnosis of a more diffuse, infiltrative pulmonary process, *i.e.*, an "open" lung biopsy. Eleven patients also underwent bronchoscopy as part of their preoperative workup, without definitive diagnosis in 82% (9 of 11 patients). Percutaneous needle biopsy, using either fluoroscopic or computed tomographic control, was completed in 10 patients with a nondiagnosis rate of 80% (8 of 10 patients).

Forty-five of the 55 patients underwent thoracoscopy for diagnosis of a solitary lung nodule or mass. The lung lesion was an isolated finding and was initially discovered by a routine chest x-ray examination or as part of the evaluation of new respiratory symptoms. Treatment for those proved to have non-small cell lung cancer was effected following successful diagnostic thoracoscopy. The other 10 patients presented with single or multiple nodules identified in the evaluation or follow-up of patients with known malignant disease. Nine patients had previous treatment for neoplastic disease including lung, breast, and colon cancer, malignant melanoma, Wilms tumor, and meningioma. One patient had a clinical stage I nonsmall cell lung cancer of the right upper lobe and a contralateral, undiagnosed 6-mm nodule. Figure 2 provides the radiographic appearance of this patient and is illustrative of the type of small, peripheral nodule that was present in most of our patients.

### RESULTS

The diagnoses that were obtained are listed in Table 1. Seventeen of the 45 patients with solitary pulmonary nodules were eventually proven to have a malignant etiology, and 28 had a benign diagnosis established. Of the 10 patients with known malignant disease, and new single or multiple nodules, metastatic disease was proven in 6 patients, nonsmall lung cancer in 2, a carcinoid in 1, and a benign diagnosis in 1 patient. There were no false-positive results, but there was one missed lesion. This patient had a 1.3 cm, intraparenchymal nodule. A blind thoracoscopic wedge resection was performed after placement of a guide wire in the radiology department. No lesion was found in the removed tissue and a repeat, successful thoracoscopy was required.

There were few complications. A total of 10 patients required conversion to an open thoracotomy. In 7, this was due to the inability to achieve adequate exposure or the presence of dense adhesions, preventing full evaluation of

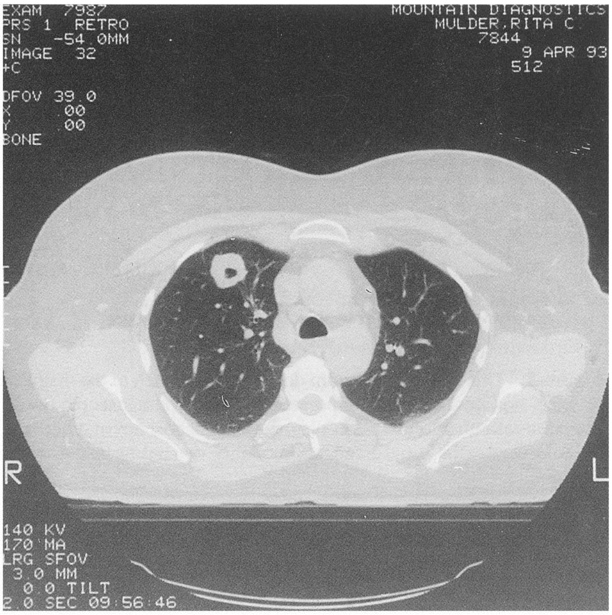

**Figure 2** A. This chest CAT scan demonstrates a right upper lobe nonsmall cell lung cancer.

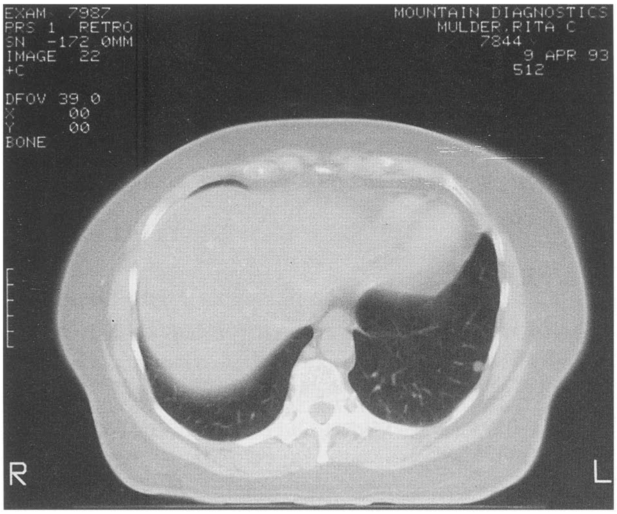

**Figure 2** B. This chest CAT scan at a lower level in the same patient demonstrates a peripheral, small left lung nodule. This nodule was removed by thoracoscopic wedge resection and was proven to be a primary carcinoid tumor. The patient went on to a curative right upper lobectomy.

the pleural cavity. Two tumors were too large to remove through an intercostal incision. Portal insertion and/or intrapleural dissection caused a bronchial tear in one patient and a thoracotomy was required for an open repair. One patient suffered a late postoperative pneumothorax, following removal of the chest tube placed during surgery. One patient required prolonged intubation and ventilatory support following thoracoscopy, but made a full recovery. There was only one inpatient death, in a patient with metastatic cancer, which was due to multiple organ system failure.

Ten patients underwent a standard anatomic resection (3 pneumonectomy, 7 lobectomy) for nonsmall cell lung cancer through an open thoracotomy during the same anesthetic as the diagnostic VATS wedge resection. In contrast, another four patients elected to await final, permanent section analysis of the wedge resection specimen before making a final commitment to surgical resection.

### DISCUSSION

The value of endoscopic or minimally invasive surgery is well documented for disease of the abdominal cavity. Laparoscopic cholecystectomy is state-of-the-art, with many other procedures becoming commonplace. Conventional thoracoscopy has been used for evaluation of pleural disease for years (Weissburg and Kaufman). With the advent of video technology, the utilization of VATS for performance of intrathoracic procedures is rapidly expanding. The role of VATS in the care of lung cancer patients, which could include diagnostic, staging, and/or therapeutic maneuvers, is presently incompletely defined.

Staging procedures would seem to lend themselves to thoracoscopy. Biopsy of aortopulmonary lymph nodes, subcarinal, and hilar lymph nodes is possible and may be shown to be complementary to the use of CAT scanning and cervical mediastinoscopy. Increasing the accuracy of clinical staging of intrathoracic disease will aid the therapeutic decision-making process by identifying or ruling out N1, N2, and N3 lymph node disease.

Wedge resection of lung cancers has been shown to be suboptimal treatment for nonsmall cell lung carcinoma. Excessively high local recurrence rates following even open wedge resection demonstrates the necessity for resection along anatomic lines (Ginsburg, personal communication) Lobectomy with a VATS technique has not yet been established as either a safe or an adequate cancer operation, as thoracoscopic treatment of nonsmall cell cancer by anatomic resection is limited by the challenge of dissecting and controlling vascular structures and the necessity of a generous incision to permit specimen removal. (Lewis *et al.*). Accordingly, thoracoscopic approaches are not now generally believed to be ready to be considered proven surgical alternatives for curatively intended surgical treatment of nonsmall cell lung cancer.

We evaluated the value of VATS as it relates to diagnosis. There are multiple aspects to this. In the case of a solitary pulmonary nodule, VATS wedge resection provides the entire specimen for histologic examination, thus establishing a certain diagnosis by eliminating the possibility of a false negative result. If nonsmall cell lung cancer is found, definitive surgical resection can be performed via a standard thoracotomy at the same time or can be deferred. If a benign diagnosis is established, no further cancer follow-up is required and the patient's peace of mind is assured. This benefit is achieved without the necessity of a thoracotomy. We are able to establish a definitive diagnosis in 44 of 45 patients, an accuracy rate of 98%. This allowed institution of definitive therapy in the cancer patients and definitely excluded cancer, and worry, in the other patients.

The diagnostic value of both bronchoscopy and fine needle aspiration biopsy (FNAB) are well established and both will continue to have an important role. The majority of our patients had small, peripherally located pulmonary nodules which accounts for the low diagnostic yield of these procedures in this group. We propose that VATS should be used as a standard part of the armamentarium for diagnosis of solitary pulmonary nodules. As outlined in Figure 3, patients should start with the standard chest x-ray and CT scan for staging of their disease. VATS should usually follow a nondiagnostic bronchoscopy or FNAB. Alternatively, VATS can be considered as the initial and definitive diagnostic method, especially for peripheral lesions. Either way, the value of thoracoscopy is to make a definitive diagnosis.

Our experience demonstrates another diagnostic role for VATS. That is, for evaluation of single or multiple lung nodules in patients with known cancer. Again, the principal benefit is obtaining the entire specimen for histologic examination without having to expose the patient to the discomfort, morbidity, and mortality of a standard thoracotomy. FNAB is, again, a reasonable way to begin the evaluation in these patients. When it is not diagnostic or when the yield of FNAB can be predicted to be low, such as in our patient illustrated in Figure 3, then VATS for definitive wedge resection is appropriate.

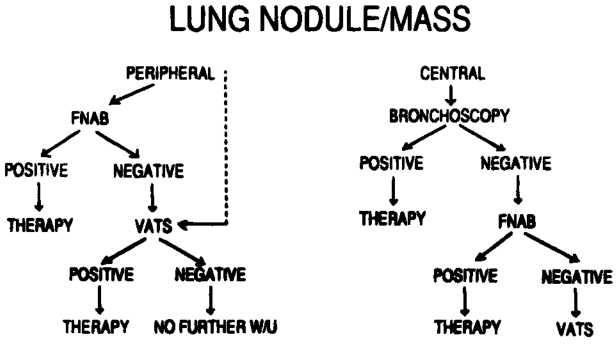

**Figure 3** This clinical algorithm depicts an approach to the diagnosis of lung nodules which incorporates VATS. Thoracoscopic wedge resection can either follow a non-diagnostic bronchoscopy and/or FNAB or can be used as the initial diagnostic modality, depending on the clinical situation.

At present, thoracoscopic identification and biopsy of pleural based nodules is more straightforward than location and removal of deeper seated, intraparenchymal nodules. Many groups are now working to establish reliable methods to solve this challenge, such as intraoperative ultrasound, radiologic fine needle placement, or methylene blue staining of the overlying lung. With experience, patience, and the utilization of these sophisticated localization techniques, the ability to find and remove these buried nodules will improve.

VATS is a versatile technique. Its safety and efficaciousness, which will only improve over time, argue for its routine use in the diagnosis of lung nodules.

### REFERENCES

Ginsburg R. J., and the Lung Cancer Study Group. Personal communication

Lewis R. J., Sisler G. E., Caccavale R. J.: Imaged thoracic lobectomy. Should it be done? *Ann Thor Surg* 1992;54:80–83

Weissberg D., Kaufman M.: Diagnostic and therapeutic pleuroscopy. *Chest* 1980;78:732–735

